# Agent Transparency, Situation Awareness, Mental Workload, and Operator Performance: A Systematic Literature Review

**DOI:** 10.1177/00187208221077804

**Published:** 2022-03-11

**Authors:** Koen van de Merwe, Steven Mallam, Salman Nazir

**Affiliations:** 67034 DNV, Høvik, Norway; 7928 University of South-Eastern Norway, Borre, Norway; 7928 University of South-Eastern Norway, Borre, Norway

**Keywords:** PRISMA, human-automation interaction, automation transparency, information disclosure, seeing into

## Abstract

**Objective:**

In this review, we investigate the relationship between agent transparency, Situation Awareness, mental workload, and operator performance for safety critical domains.

**Background:**

The advancement of highly sophisticated automation across safety critical domains poses a challenge for effective human oversight. Automation transparency is a design principle that could support humans by making the automation’s inner workings observable (i.e., “seeing-into”). However, experimental support for this has not been systematically documented to date.

**Method:**

Based on the PRISMA method, a broad and systematic search of the literature was performed focusing on identifying empirical research investigating the effect of transparency on central Human Factors variables.

**Results:**

Our final sample consisted of 17 experimental studies that investigated transparency in a controlled setting. The studies typically employed three human-automation interaction types: responding to agent-generated proposals, supervisory control of agents, and monitoring only. There is an overall trend in the data pointing towards a beneficial effect of transparency. However, the data reveals variations in Situation Awareness, mental workload, and operator performance for specific tasks, agent-types, and level of integration of transparency information in primary task displays.

**Conclusion:**

Our data suggests a promising effect of automation transparency on Situation Awareness and operator performance, without the cost of added mental workload, for instances where humans respond to agent-generated proposals and where humans have a supervisory role.

**Application:**

Strategies to improve human performance when interacting with intelligent agents should focus on allowing humans to see into its information processing stages, considering the integration of information in existing Human Machine Interface solutions.

## Introduction

The human factors community has long had an interest in understanding the interactions between humans and automation, that is, the tasks executed by a machine agent of a function previously performed by a human (Parasuraman & Riley, 1997; Rasmussen, 1983). Central topics of research include understanding the benefits and concerns of replacing humans with automation (e.g., Bainbridge, 1983; Strauch, 2018), the need for appropriate design of automation (Norman, 1990), the effect of automation failures on human take-over responses (Endsley & Kiris, 1995), factors pertaining to automation use, disuse, and misuse (Parasuraman & Riley, 1997), human performance in taking over from automation (Eriksson & Stanton, 2017; Hergeth et al., 2017; Weaver & DeLucia, 2020), and the consequences of levels of automation on Situation Awareness (SA), mental workload, and operator performance (Endsley & Kaber, 1999; Jamieson & Skraaning, 2020; Onnasch et al., 2014). Combined, these studies culminate to the notion of an automation conundrum (Endsley, 2017), which is the problem that the more reliable and robust automation becomes, the less likely it is that a human supervisor will notice critical information and will be able to effectively intervene when required. This problem may be exacerbated with advanced automation or intelligent agents able to function independently, but still require human supervision. Considering the rapidly developing and ubiquitous presence of technology in our society, there is an urgent and continuous need of research into understanding and enhancing interactions between humans and automation such that collaboration and performance can be supported (Hancock et al., 2013; O’Neill et al., 2020; Warden et al., 2019).

### Automation and Agents

The terms “automation” and “agent” are used interchangeably in the literature. For example, Lee and See define automation as “technology that actively selects data, transforms information, makes decisions and controls processes” (2004, p. 1). Parasuraman and Riley define automation as “the execution by a machine agent (usually a computer) of a function that was previously carried out by a human” (1997, p. 231). Rao and Georgeff (1995) describe a rational agent as one having certain “mental attitudes of Belief, Desires and Intention (BDI), representing, respectively, the information, motivational, and deliberative states of the agent” (1995, p. 1). In AI, the term “intelligent agent” refers to an autonomous entity having goal-directed behavior in an environment using observation through sensors and execution actions through actuators (Russell & Norvig, 2022). Examples of the application of agents can be seen in the automotive industry (Society of Automotive Engineers, 2021), healthcare (Coronato et al., 2020; Loftus et al., 2020), unmanned aerial vehicles (UAV) (Hocraffer & Nam, 2017), manufacturing (Elghoneimy & Gruver, 2012), and recent development towards maritime autonomous surface ships (IMO, 2018). Even though agents can be very sophisticated and can perform certain task with a high degree of independence, they often require some form of human supervision in case of failures or unforeseen situations. However, human supervision of such agents may pose challenges as AI behavior and reasoning can be difficult or even impossible to understand for humans (Doshi-Velez & Kim, 2017; Lipton, 2017). Still, to enable interaction between humans and agents, a system component capable of handling human-machine interactions is typically deployed, that is, the Human Machine Interface (HMI). The HMI supports human-machine interactions by providing relevant feedback to support SA and by allowing for appropriate input commands to support action execution.

Norman (1990) has previously advocated the use of appropriate feedback when interacting with automation, arguing that the problem with keeping humans in the loop is not necessarily automation itself, but the lack of adequate information provided to them. Likewise, Christoffersen and Woods (Christoffersen & Woods, 2002) have discussed the need for systems to be observable to humans to enhance human-agent collaboration. That is, providing feedback to the operator in terms of its changes to the agent’s current state (events) allows for anticipatory reasoning (future states) and for quick detection of abnormalities through pattern recognition. Also, Lee and See (2004) argued for a number of elements that should be conveyed to the user, such as showing the automation’s purpose, past performance, and its processes and algorithms. In addition, intermediate internal process results should be shown that are understandable to the operator in a simplified way. Also, the purpose, design basis, and range of application should be conveyed that relate to the user’s goals. Supplying this information to the operator would result in appropriate reliance and trust in the automation. Hence, when humans interact with agents, the HMI can be used to convey the agent’s state, its modes, and limitations, and provide understandability and predictability regarding its current actions and future actions, that is, providing “transparency” to its user (Endsley, 2017).

### Transparency

There are two common interpretations of agent transparency found in the literature: “seeing-through” and “seeing-into” (Ososky et al., 2014; Sheridan & Verplank, 1978; Skraaning et al., 2020). The “seeing-through” interpretation states that automation should be designed in such a way as to appear invisible to its user. For example, in teleoperation using robots, transparent automation, for example, through means of low latency devices, effective feedback mechanisms, and immersive HMIs, allows an operator to perceive and manipulate the environment as if there was no automation in between. In this case, the automation is purposefully made invisible to the user allowing for enhanced awareness and “presence” of the remote environment. Conversely, the “seeing-into” interpretation aims to make the automation or agent visible to the human to allow for enhanced understanding of the agent itself. In this case, the agent is made transparent, or better: “apparent” (Sheridan & Verplank, 1978; Skraaning et al., 2020), to its user by purposefully conveying what it is doing, why it is doing it, and what it will do next. In this perspective, transparency is an HMI design principle applied to the technology, based on the notion that information from and about the agent is directly observable to the user. In this paper, we will adopt the “seeing-into” perspective when referring to transparency.

Transparency information should allow for a user to “see into” the agent and better understand its inner processes, thereby enhancing the user’s ability to assess the agent’s performance and knowing when to manually take-over or not. Conversely, a lack of “transparency” (Endsley et al., 2003), “observability” (Christoffersen & Woods, 2002), “interpretability, explainability and predictability” (Hepworth et al., 2020), or “affordance” (Chen et al., 2014) of the agent may make it difficult for an operator to grasp what it is doing, why it is doing it, and what it is going to do next. This, in turn, may lead to poor decision making regarding when to use (and when not use) automation (Beck et al., 2007; Endsley & Kiris, 1995; Parasuraman & Riley, 1997). As such, exposing the inner workings of the automation to its human supervisor should, at least theoretically, enhance the operator’s performance.

### Transparency and Human Performance

Recent publications have explored evidence regarding automation transparency, that is, “seeing-into.” Bhaskara et al. (2020) identified and compared the dominant transparency models in the contemporary literature: Human-Robot Transparency Model (Lyons, 2013); Situation-Awareness Agent-based Transparency model (SAT; Chen et al., 2014). For these models, the authors reviewed five experimental studies that implemented transparency across a range of tasks and domains. Results from key human factors variables, including operator performance, SA, and mental workload indicated that there is emerging evidence regarding accurate use of automation with increased transparency, potential evidence for its effect on SA and a potential cost in terms of mental workload, as measured through pupil diameter in one study (Wright et al., 2017). However, results were not consistent in terms of the correlation between the degree of transparency and performance variables. In other words, more transparency did not consistently produce improved operator performance outcomes. Hence, the effect of transparency may be dependent on other factors such as context and information type.

In a similar review, Rajabiyazdi and Jamieson (2020) reviewed the experimental evidence for four transparency models: Human-Robot Transparency Model (Lyons, 2013); (Dynamic) Situation-Awareness Agent-based Transparency model (SAT; Chen et al., 2014, DSAT; Chen et al., 2018); and the Coactive System Model based on Observability, Predictability, and Directability (Johnson et al., 2014). Five experimental studies were reviewed for their empirical evidence, of which two studies overlapped with Bhaskara et al. (2020). The authors concluded that the validation efforts for the transparency models have been largely incomplete or have provided inconclusive evidence. For example, there were differences among the studies in how the SAT model was interpreted and operationalized, that is, what level of transparency relates to which type of information, potentially leading to differences in outcomes. Also, even though some of the studies were based on the same theoretical model and applied in a similar context, they yielded inconsistent human performance outcomes in terms of SA, workload, and operator performance, amongst others. Nevertheless, considering the continuous development of advanced automation, the authors concluded that there is an ongoing and increasing need to further understand the means with which to convey its inner workings to the operators and assess its effect on human factors variables.

### This Study

This review aims to expand on the evidence base for automation transparency and operator performance by focusing on a broader body of literature beyond those studies discussed in the reviews mentioned earlier. This is to be achieved by taking the original concept of transparency as the starting point for the review regardless of the transparency model. As the concept of “seeing-into” transparency is about conveying the inner workings of the automation to provide understandability and predictability about its actions, a broader scope may reveal additional insights not captured by model-specific studies (Bhaskara et al., 2020; Rajabiyazdi & Jamieson, 2020). This approach may uncover other studies not included in the abovementioned reviews that nevertheless provide evidence for the relationship between transparency and central human factors variables: SA, mental workload, and operator performance. These variables were chosen because information disclosure to reveal the inner workings of an agent is closely linked to the operator’s mental picture of the agent’s present and future state. As such, if the agent can convey to the user which information it is presently processing, how it is processing it, and what its future state will be, this would suggest that this information would have a positive effect on operator SA (Endsley, 1988, 1995). However, because transparent automation provides “understandability and predictability of actions” to a human operator (Endsley, 2017; Endsley et al., 2003), the HMI between the agent and the operator is often manipulated to allow for this. As mental workload concerns the allocation of limited internal resources in meeting external demands (Hancock et al., 2021), adding information increases the amount of information required to build and maintain SA, potentially requiring additional cognitive effort (Chen et al., 2014, 2018; Helldin et al., 2014). On the other hand, it may also be reasoned that assessing the performance of an agent is facilitated when information about the agent is made directly available to the user compared to when it is not (Chen et al., 2018). As such, the consequences of transparency information for mental workload may be mediated by other factors than amount of information only, for example, display design (Li et al., 2020; Vicente, 2002). Nevertheless, as transparent automation should allow an operator to better assess the agent’s performance, that is, its reliability, predictability, and ability (Lee & See, 2004), it should also improve the operator’s ability to perceive, comprehend and project the performance of the agent and thereby deciding whether to use the automation or not (Beck et al., 2007; Parasuraman & Riley, 1997). This potential “free lunch” (Wickens, 2018), that is, the ability of transparency to alleviate some of the effect of the automation conundrum without reducing automation’s benefit, warrants a further and systematic focus.

## Method

This study uses the Preferred Reporting Items for Systematic review and Meta-Analysis protocol (PRISMA) as a basis for the systematic literature review (SLR; Moher et al., 2009, 2015). The PRISMA protocol provides a pre-defined and structured methodological approach to literature reviews including its data gathering, analysis, and reporting. Using a pre-defined approach reduces the potential for bias and enhances clarity, auditability, replicability, and transparency of the review (Booth et al., 2016). In brief, the PRISMA protocol uses a three-step approach starting with searching for relevant literature in relevant databases using a specified search string where the literature data is screened based on a pre-defined set of eligibility criteria. Second, an in-depth assessment is performed based on a review of the full texts generating a final dataset of literature. And finally, this dataset is analyzed as part of the qualitative data analysis.

### Database Search and Data Screening

The following inclusion criteria were established for the initial screening of the literature sample. First, only peer-reviewed studies published between the 1st of January 2000 and the 5th of January 2021 (the sample date) were considered. Second, studies that describe transparency effects on operator performance using experimental studies as a data source were considered.

The following exclusion criteria were established for the initial screening. First, non-English articles, articles from outside the time-period, non-peer reviewed, or gray literature (i.e., white papers, books, technical reports, book chapters, posters), and articles that not explicitly address automation transparency in experimental studies.

For screening the full-text literature, the following inclusion criteria were used. First, this SLR was interested in studies presenting primary data that compared degrees of implementation of transparency in terms of SA and/or mental workload and/or operator performance metrics. Second, studies were considered if they met all the following characteristics based on the PICOC criteria (Booth et al., 2016; Petticrew & Roberts, 2006):

• Population: Users in the safety critical domain
• Intervention: Application of transparency in automation design
• Comparison: Comparing degrees of transparency
• Outcomes: The studies reported on SA, and/or mental workload, and/or operator performance metrics as dependent variables
• Context: The studies reported on findings obtain from a simulated- (experimental) and/or operational environment

To obtain the dataset, relevant databases were chosen based on their publication scope within the domains of psychology, technology, and engineering. The chosen databases were Scopus (with ScienceDirect for the full-text journals), IEEE Xplore, and Web of Science and were sampled using a search string.

The search string contains three components: the object of interest (e.g., automation), its characteristics (e.g., transparency), and its effect on operators (e.g., behavioral indicators and psychological constructs). The search aimed to balance breadth and depth of the field, and therefore the search was based on keywords only. The following search string was used in each of the chosen databases:

(Autom* OR Autonom* OR Robot OR Machine OR Agent)

AND

(Transparen* OR Observab* OR Explainab* OR Afford*)

AND

(“Operator performance” OR “Human performance” OR “Situation Awareness” OR Workload OR Effectiv*)

**Figure 1.**
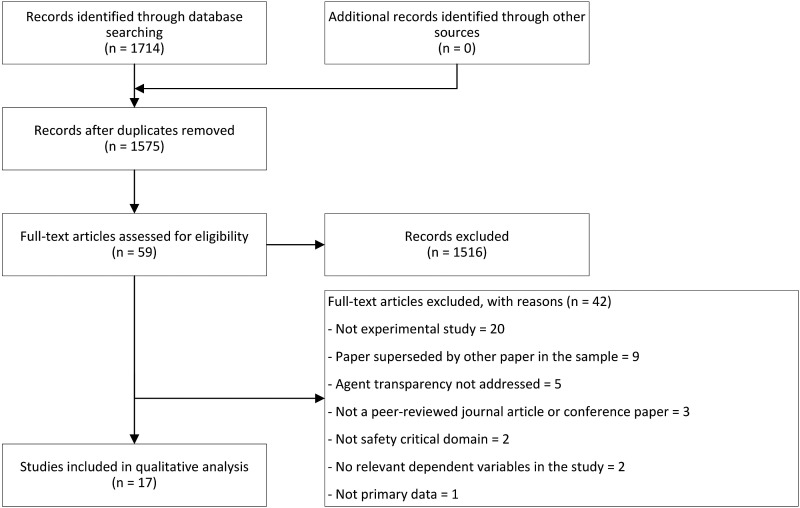
Flow diagram of the study selection based on the PRISMA protocol.

Figure 1 provides the process and results of the database search. The search resulted in a combined sample of 1714 articles of which there were 139 duplicates. Based on the sample of 1575 papers, the initial screening was performed based on the eligibility criteria described above. This consisted of a review of the titles and abstracts against the criteria. When in doubt, the paper was kept for full-text review. This resulted in a reduced sample of 59 articles for full-text review.

### Full-Text Review

The full-text review was performed by the first author based on the full-text eligibility criteria. A subset of 25 full-text papers out of the 59 papers were reviewed independently by the other authors. The results from this independent review of papers were cross verified with the results of the first author in a workshop. Any disagreements were resolved, and reasons for exclusion were noted. Of the full-text sample of 59 papers, 42 papers were excluded with reasons based on the pre-defined criteria (see Figure 1). As such, a final dataset of 17 full-text articles remained for inclusion in the qualitative analysis: 11 journal articles and six conference papers.

### Data Extraction and Analysis

Data from each individual study from the final dataset was extracted including the domain in which transparency was studied, the sample size, which (if any) transparency model was used, the Human-Automation Interaction type (HAI), how transparency was operationalized, and the comparisons that were made in the experimental study (see Table 1). For each of the studies the results were extracted, including SA effects of using the automation in the study, the effect on mental workload, and the behavioral/performance measures employed in the study (see Table 2).

**TABLE 1.** Characteristics of the Studies

Reference	Domain, *N*	Model	HAI Type	Operationalization of Transparency and Comparisons
Mercado et al. (2016)	Military UAV	*SAT model:* The visualization of the agent’s current action and plans (Level 1), its reasoning and constraints (Level 2), and its projected outcomes and uncertainties (Level 3).	*Respond to proposals:* Monitor and control multiple unmanned vehicles (land, sea, air vehicles) and evaluate proposed plans (A or B) by an intelligent agent based on speed, coverage, and capability.	*Level 1:* Basic plan information provided by indicating which unmanned vehicles were in use and which paths they used.
	30 Non-SME			*Level 1+2:* Level 1 plus the agent’s reasoning and rationale behind recommending the plans was provided via a text box and sprocket graphic.
				*Level 1+2+3:* Level 1+2 plus projection of uncertainty information related to a successful outcome. Uncertainty was presented through the opacity of vehicle icons, road colors, sprocket graphic wedges, and bullet points in the text box
Roth et al. (2020)	Military UAV	*SAT model:* The visualization of the agent’s current action and plans (Level 1), its reasoning and constraints (Level 2), and its projected outcomes and uncertainties (Level 3).	*Respond to proposals:* Perform mission planning and system management of a manned-unmanned teaming operation (manned helicopter + unmanned aerial vehicle). Execute a helicopter transport mission with take-off, transit, and landing.	*Low transparency:* Level 1 information only. For mission planning the automation’s goal, settings, and level of automation were displayed. For system management an “Adopted Tasks”-list was shown.
	10 Non-SME		In mission planning, the participants had to evaluate the validity of the planning proposals performed by the agent and find violations.	*High transparency:* Level 1+2+3 information only. For mission planning the automation’s goal, settings, and level of automation (Level 1), symbols representing the events that justified an intervention (Level 2), a timeline presenting the temporal outcomes projected by the agent (Level 3) were shown. For system management an “Adopted Tasks”-list (Level 1), “Critical Events”-list, “Neglected Tasks”-list, and “Current Load”-indicator (Level 2), and “To Do Tasks”-list and a timeline presenting the predicted future workload (Level 3) were shown.
			In system management, the participants performed the role as pilot-flying in the helicopter. Participants were tasked with monitoring and evaluating the agent’s assistance. Flight control were not part of the tasks.	
Stowers et al. (2020)	Military UxV	*SAT model:* The visualization of the agent’s current action and plans (Level 1), its reasoning and constraints (Level 2), and its projected outcomes and uncertainties (Level 3).	*Respond to proposals:* Monitor and control multiple unmanned vehicles (land, sea, air vehicles) and evaluate proposed plans (A or B) by an intelligent agent based on speed, coverage, and capability.	*Level 1+2:* Level 1 and 2 information was displayed through the size of the unmanned vehicles’ icons with larger icons depicting the faster unmanned vehicles.
	53 Non-SME		See also Mercado et al. (2016) in this table.	*Level 1+2+3:* Level 1+2 plus level 3 information displayed by an icon attached to the unmanned vehicles indicating the time it was from its goal location.
				*Level 1+2+3+U:* Level 1+2+3 plus uncertainty information displayed through changes in opacity of the unmanned vehicles’ icons.
Bhaskara et al. (2021)	Civil UxV	*SAT model:* The visualization of the agent’s current action and plans (Level 1), its reasoning and constraints (Level 2), and its projected outcomes and uncertainties (Level 3).	*Respond to proposals:* Perform and complete unmanned vehicle control missions by selecting the most appropriate plan (A or B) against mission attributes. Participants were assisted by automation that based its decision on a formula taking into account time to search area, search time, and fuel consumption.	*Level 1:* The automation evaluated each unmanned vehicle’s capabilities against the weighted mission attributes to determine and display the most suitable plan. No copy of the automation’s formulae was provided.
	176 Non-SME			*Level 1+2:* As per Level 1. In addition, participants were informed of the automation’s formulae and had a hard copy of these.
				*Level 1+2+3:* As per Level 1+2. In addition, participants received a visualization of the relative capability projection of the unmanned vehicles associated with Plans A and B (blue shaded bars presented on the interface).
Göritzlehner et al. (2014)	Air Traffic Control	*Non-specific:* Visual representation of a specific automated resolution advisory within the solution space for air traffic (i.e., go or no-go areas in speed and heading for an aircraft).	*Respond to proposals:* Ensure conflict free traffic in a free-flight Air Traffic Control scenario. Respond to resolution advisories by the automation by accepting or rejecting. Rate the agreement with the advisory.	*Transparency 1:* No support provided. The Solution Space Diagram was turned off.
	12 Non-SME			*Transparency 2:* Support provided by showing heading bands, indicating unsafe heading regions.
				*Transparency 3:* Support provided by showing triangle-shaped conflict areas, indicating unsafe regions in speed, and heading.
Sadler et al. (2016)	Flight planning	*Non-specific:* The provision of the rationale behind automatically derived decision recommendation in three levels of transparency: Baseline, value, and logic.	*Respond to proposals:* Land aircraft on a landing site based on recommendations from an Autonomous Constrained Flight Planner.	*Baseline:* No explanation for how the automation arrived at its recommendation was provided.
	12 SME			*Value:* Baseline plus the calculated success probability that drove the diversion recommendation was provided.
				*Logic:* Logic plus an additional explanation detailing the link between the probabilities and the information used to derive the recommendations was provided.
Guznov et al. (2020)	Robotics88 Non-SME	*SAT model:* The visualization of the agent’s current action and plans (Level 1), its reasoning and constraints (Level 2), and its projected outcomes and uncertainties (Level 3).	*Supervise automation:* Monitor and control a robot through an environment. Based on a video feed and messages from the robot, participants could intervene if the robot was believed to be off the path.	*Level 2:* The robot’s text message informed the participant of its actions and the reasoning processes behind them. For example, when the robot would approach a turn, it would report: “I see an obstacle on the right, so I’m turning left.”
				*Level 3:* Level 2 plus its future states. For example, when the robot approached a turn, one of the multiple messages it reported was: “I see an obstacle on the left, so I will turn right in order to avoid collision.”
Chen et al. (2014)	Military UAV	*Non-specific:* Visualization of the implications of the level of automation on the unmanned aerial vehicle’s autonomy capability to the operator through symbols and colors.	*Supervise automation:* Supervise a group of four unmanned aerial vehicles with various functional levels of automation in their search zones. Perform a search operation in the zones.	*Non-transparent HMI:* The unmanned aerial vehicle did not provide information regarding changes to its current and projected flight path.
	43 Non-SME			*Transparent HMI:* The unmanned aerial vehicle provided visual information regarding changes to its flight path.
Sanders et al. (2014)	Ground troops support	*Non-specific:* The amount of information that the system provides to the user about its internal operations (i.e., explaining why it behaves as it does).	*Supervise automation:* Control a soldier to find civilians and mark their location on a map. Also, assist an autonomous squad member (i.e., robot) by responding to questions (i.e., make navigational decisions).	*Minimal:* The autonomous squad member provided a short, three-word description of the problem it encountered.
	73 Non-SME			*Contextual:* The autonomous squad member provided the decision it was requesting and a small amount of information about the situation to help the user make the decision.
				*Constant:* In addition to the above, the autonomous squad member provided the user with a constant stream of information.
Chen et al. (2015)	Military UAV	*Non-specific:* Visualization of autonomy and functional capabilities of the agent through a (textual) natural language dialogue.	*Supervise automation:* Monitor and control four unmanned aerial vehicles (in various levels of automation) in transit mode, respond to a hazardous event with an avoidance maneuver (hazard avoidance mode), and perform a search activity (search mode).	*Limited transparency:* No communication mechanism in place to allow the sharing of status between the unmanned aerial vehicles and participant.
	36 Non-SME			*Increased transparency:* A message dialogue box was in place to allow communication to be established between the unmanned aerial vehicles and the participant.
Skraaning & Jamieson (2021)	Nuclear16 SME	*Non-specific:* The observability of responsibilities, capabilities, goals, activities, and/or effects of automation in the human system interface.	*Supervise automation:* Control a simulated nuclear power plant and deal with minor to major system upsets including taking corrective action.	*Traditional:* The human system interface did not provide explicit visual or verbal feedback about automation responsibilities, capabilities, goals, activities, and/or effects. Operator had to infer these attributes of automation from changes in the plant process as reflected in a conventional human system interface for supervisory control.
				*Transparent:* The interface provided explicit verbal and visual information about automation activities to the operators. Key automatic devices on a large-screen overview display, dedicated displays for detailed monitoring, display of tracking of automation sequences, and verbal feedback about the activity of automatic systems from automation were used.
				Verbal feedback was provided each time automatic devices started or failed. The information was limited to behavioral feedback from automation (what happened) announced repeatedly.
	Nuclear	As above.	As above.	As above, and verbal feedback was provided only when the executive automatic programs started, or automatic devices failed. The feedback from automation was both behavioral (what happened) and diagnostic (why it happened) and was announced once.
	18 SME			
	Nuclear	As above.	As above and monitor automatic scripts responsible for performing a cold start of the plant to 50% reactor power. When the automatic procedure paused (e.g., due to a technical failure) intervene by assuming manual control, restarting automation or shutdown.	*Traditional:* No explicit information regarding the information about the activities of the plant-wide procedure automation. Operators had to derive this from the process events and changes to system states.
	27 SME			*Transparent:* Explicit information about automation status and actions provided through a dedicated overview display showing the automation’s progress. Color coding was used to depict status including a detailed list of procedural steps. The detailed automation display depicted historical and ongoing automation activities.
Selkowitz et al. (2015)	Search & Rescue45 Non-SME	*SAT model:* The visualization of the agent’s current action and plans (Level 1), its reasoning and constraints (Level 2), and its projected outcomes and uncertainties (Level 3).	*Monitor automation:* Monitor an autonomous squad member as it moves through an urban area. The autonomous squad member moved on its own accord taking into account obstacles and dangers. Its route was revealed from waypoint to waypoint by a navigation line.	*Level 1:* The autonomous squad member provided its current location, its route, and its current resources.
				*Level 1+2:* Level 1 plus the autonomous squad member provided its affordances and hazards it encounters during its task execution.
				*Level 1+2+3:* Level 1+2 plus the autonomous squad member provided its environmental constraints with their associated uncertainties and its predicted resources at the end of the mission.
Selkowitz et al. (2017)	Military UAV	*SAT model:* The visualization of the agent’s current action and plans (Level 1), its reasoning and constraints (Level 2), its projected outcomes (Level 3), and its uncertainties (Level 3+U).	*Monitor automation:* Monitor a simulated environment for threats and mark these on the display. Monitor an autonomous squad member’s display for its decisions.	*Level 1:* The autonomous squad member provided its current resource levels, its understanding of the squad’s current status, its understanding of the environment around it, the current highest influence on its motivator (e.g., time), and its current action/position.
	60 Non-SME			*Level 1+2:* Level 1 plus the autonomous squad member’s reasoning behind its current action (e.g., a clock icon).
				*Level 1+2+3:* Level 1+2 plus the projected outcomes of its current actions and reasoning (e.g., projected time displayed).
				*Level 1+2+3+U:* Level 1+2+3 plus the associated uncertainty for the information (e.g., time uncertainty and icon color).
Wright et al. (2020)	Ground troops support	*SAT model:* The visualization of the agent’s current action and plans (Level 1), its reasoning and constraints (Level 2), and its projected outcomes and uncertainties (Level 3).	*Monitor automation:* Monitor a video feed from a soldier team and evaluate the autonomous squad member in correctly identifying and responding to events the soldier squad encountered. Detect threats in the surrounding environment and identify events the squad encountered.	*Surface-level information:* The interface contained at-a-glance information regarding the autonomous squad member’s current actions (Level 1), the reasons for its action (Level 2), and the results of its actions (Level 3).
	56 Non-SME			*In-depth information:* Surface-level information plus additional information depicting the underlying factors that led to each specific information in the surface-level module.
Pokam et al. (2019)	Autonomous vehicles	*Human-Robot Transparency model:* The information that a robot needs to convey to a human (its intentions, tasks, analysis, and environmental constraints) and vice versa (how tasks are distributed and awareness of the human state).	*Monitor automation:* Monitor the behavior of an autonomous vehicle from the driver’s seat under different transparency conditions. No intervention was required.	*HMI 1:* The vehicle displayed no additional information about its autonomy.
	45 SME			*HMI 2:* The vehicle displayed its acquired information and its action execution.
				*HMI 3:* The vehicle displayed its acquired information, its analysis, and its action execution.
				*HMI 4:* The vehicle displayed its information acquired, its analysis, and its decision making.
				*HMI 5:* The vehicle displayed its information acquired, its analysis, its decision making, and its action execution.
Du et al. (2019)	Autonomous vehicles	*Non-specific:* The provision of explanations to justify why an action was or was not taken by the automation.	*Monitor automation:* Monitor the behavior of an autonomous vehicle from the driver’s seat. There was no need to take over control of the vehicle.	*No explanation:* The autonomous vehicle provided no explanation about its actions.
	32 SME			*After explanation:* The autonomous vehicle presented an explanation within 1 s after actions had been taken.
				*Before explanation:* The autonomous vehicle provided an explanation 7 seconds prior to its action.
Panganiban et al. (2020)	Military aviation	*Non-specific:* The intentional design of a system to communicate its capabilities and current state to support human-machine teaming.	*Monitor automation:* Fly a fighter aircraft and attack a missile site whilst cloaking the plane from detection from a nearby defensive Surface-to-Air-Missile. The autonomous wingman supported the pilot through performing surveillance (neutral condition) or additional cloaking of the pilot against the Surface-to-Air-Missile (benevolent condition).	*Neutral:* The wingman provided only information on its immediate task activities, with no additional transparency into its intentions.
	40 Non-SME			*Benevolent:* The wingman communicated its intention to support the human and to correct its errors, signaling its awareness of the human partner’s expectations.

**TABLE 2.** Study Results

Reference	HAI Type	Situation Awareness	Effect	Mental Workload	Effect	Operator Performance	Effect
Mercado et al. (2016)	Respond to proposals			Scores on NASA-TLX	↔	Correct use of proposals	↑
				Mean eye fixation duration	↔	Correct rejection of proposals	↑
				Pupil diameter	↔	Response time to proposals	↔
				Saccadic amplitude	↔		
				Saccade duration	↔		
Roth et al. (2020)	Respond to proposals	Scores on SAGAT	↑	Scores on Bedford Mental	↔	Response time to proposals	↓
		Scores on SART	↔	Workload scale		Accuracy of decisions	↔
Stowers et al. (2020)	Respond to proposals			Scores on NASA-TLX	↔	No. of correct responses	↑
						Response time	↑
Bhaskara et al. (2021)	Respond to proposals			Scores on NASA-TLX	↔	Acceptance of correct proposals	↑
				Secondary task performance (auditory recognition task)	↔	Rejection of incorrect proposals	↑
						Accuracy of automation use	↑
						Decision time	↓
Göritzlehner et al. (2014)	Respond to proposals			0-100 scale	↔	Advisory accept/reject	↔
						Agreement with advisory	↔
						Separation conflicts	↓
						Separation violations	↔
Sadler et al. (2016)	Respond to proposals					Verifications of plans	↓
						Exploring for alternatives	↔
						Agreement with plans	↔
Guznov et al. (2020)	Supervise automation	Scores on SART	↔	Scores on NASA-TLX	↑	No. of correct responses	↔
						No. of correct rejections	↔
Chen et al. (2014)	Supervise automation	Scores on SAGAT	↑	Scores on NASA-TLX	↓		
Sanders et al. (2014)	Supervise automation			NASA-TLX results not reported	?		
				DSSQ results not reported	?		
Chen et al. (2015)	Supervise automation	Scores on SAGAT	↑	Scores on NASA-TLX	↓	Initial response times for UAV to proceed on-course	↔
						Event response times for UAV to avoid hazards	↓
						Success rate in finding items	↑
Skraaning & Jamieson (2021)	Supervise automation	Scores on SART	↔	Scores on Perceived Task Complexity scale	↓	Response time to events	↓
						Detecting deviations and performing verifications	↑
						Achieving main goals	↑
		Scores on SART	↑	Scores on Perceived Task Complexity scale	↓	Response time to events	↓
						Detecting deviations and performing verifications	↑
						Achieving main goals	↑
		Scores on Process Overview Measure	↔	Scores on Perceived Task Complexity scale	↔	Detecting deviations and performing verifications	↓
						Achieving main goals	↔
						Self-rated task performance	↔
Selkowitz et al. (2015)	Monitor automation	Scores on SAGAT	↔	Scores on NASA-TLX	↔		
Selkowitz et al. (2017)	Monitor automation	Scores on SAGAT (L1 SA)	↔	Eye-fixations duration	↑		
		Scores on SAGAT (L2 SA)	↑	No. of eye fixations	↔		
		Scores on SAGAT (L3 SA)	↑	Scores on NASA-TLX	↔		
		Confidence in own L1 SA	↔				
		Confidence in own L2 SA	↑				
		Confidence in own L3 SA	↑				
Wright et al. (2020)	Monitor automation	Scores on SAGAT	↔	Scores on NASA-TLX	↔	Detecting targets	↔
						Time to identify and assess events	↔
Pokam et al. (2019)	Monitor automation	Scores on SAGAT	↔				
Du et al. (2019)	Monitor automation			Scores on NASA-TLX	↔		
Panganiban et al. (2020)	Monitor automation			Scores on NASA-TLX	↓		

Key: ↑ indicates improvement/increase, ↔ indicates that no effect was found, ↓ indicates decline/reduction, ? indicates that the outcome is unspecified, and blank cells indicate the variable was not measured.

## Results

There are multiple ways in which the data in Tables 1 and 2 can be organized and interpreted depending on specific research needs. For our analysis, we have chosen to organize the data according to human-automation interaction type. For readers interested in looking into other relations in the dataset, the tables are made available as Supplemental Material on the journal’s Web site.

### Mapping Out How Transparency Has Been Studied

Table 1 describes the characteristics of the individual papers from the data sample. Each characteristic is discussed below.

#### Research Domains

The domain which had most focus on transparency research is the military (53%), with studies primarily focusing on UAV operations and ground troops support, and one study focused on the interactions with an automated pilot flying with a human in formation (wingman). Two (12%) studies were performed in the automotive domain in relation to autonomous vehicles. The other domains in which automation transparency was researched were civil defense (12%), civil aviation (12%), nuclear (6%), and robotics (6%).

#### Transparency Models

Eight studies (47%) used the SAT model (Chen et al., 2014) as a basis for the design of the automation. The studies that employed this model typically used the various levels described by the model to develop user interfaces that provide users with relevant transparency information. For example, Selkowitz et al. (2017) developed a user interface showing an autonomous squad member’s current resource levels (Level 1), prioritizations when following the squad (Level 2), consequences on future resource levels (Level 3), and the uncertainties related to this information. The other studies from the sample that used the SAT model have developed interfaces based on a similar approach (Bhaskara et al., 2021; Guznov et al., 2020; Mercado et al., 2016; Roth et al., 2020; Selkowitz et al., 2015; Stowers et al., 2020; Wright et al., 2020).

One study (6%) used Lyons’ Human-Robot Transparency model (2013). Lyons describes the need for sharing information from the automation to the human (robot-to-human factors), as well as from the human to the automation (robot-of-human factors). Hence, Lyons’ transparency model focuses on the requirements to the automation’s information provision to the user, as well as the automation’s capability to understand the human. Pokam et al. (2019) applied this model to develop the interface for an automated driving solution showing the conditions for when autonomous mode was available, understand the actions by the vehicle, why a given maneuver was carried out and showing what the automation perceived in order to understand its analyses and decisions.

Eight studies (47%) were not limited to a single transparency model but used various transparency sources as the basis for automation design. For example, Skraaning and Jamieson (2021) stated that the automation displays that were used in their nuclear control room study were designed “with the transparency principle in mind” (2021, p. 380). They define transparency as “the design principle that the responsibilities, capabilities, goals, activities and/or effects of automation should be directly observable in the [Human System Interface]” and refer to Norman (1990), Christoffersen and Woods (2002), Johnson et al. (2014), and others as their inspirational sources. Likewise, Du et al. (2019) focused on explanations provided by the automation as a means to expose users “to the inner workings or logic used by the automated system” (2019, p. 429). Also, Chen et al. (2014, 2015), Sanders et al. (2014), Göritzlehner et al. (2014), Sadler et al. (2016), and Panganiban et al. (2020) have used various transparency sources as inspiration for their automation design.

The dataset did not include experimental studies for the Coactive System Model based on Observability, Predictability, and Directability (Johnson et al., 2014).

#### Human-Automation Interaction Type

In six studies (35%) participants were tasked with *responding to proposals* provided by the automation. Mercado et al. (2016) and Stowers et al. (2020) performed similar experiments where participants were asked to monitor and control multiple unmanned vehicles (land, air, and sea; UxV) in a base-defense task. An intelligent agent generated proposals on how to best defend the base based on speed, coverage, and capabilities of the unmanned vehicles. The participants were required to choose the most optimal plan. Similarly, Bhaskara et al. (2021) required participants to select the best unmanned vehicle to perform a task. Participants were assisted by a system that provided two plans with regards to which unmanned vehicle was most capable based on its time to reach a search area, search time needed and fuel consumption. The participants were asked to check the accuracy of the proposals against a set of criteria and choose the best one. Roth et al. (2020) also required participants to check the validity of the agent’s proposals and find violations to previously given constraints for an UAV mission. Participants in the experiment by Göritzlehner et al. (2014) took the role of an air traffic controller and were tasked with ensuring conflict-free traffic in a simulated airspace. The automation provided advisories to resolve conflict situations, and the participants were required to either accept or reject these based on their perception of the situation. Finally, Sadler et al. (2016) used airline pilots in the role of enhanced ground operators that were required to land aircraft at alternative landing sites when their primary destination was unavailable. An Autonomous Constrained Flight Planner was used to provide the operators with recommended diversions which they were asked to check for its validity.

Five studies (30%) required participants to *supervise the automation* (i.e., monitor, respond to, and manually operate) when required. In three separate experiments, Skraaning and Jamieson (2021) required licensed operators to monitor, control, and operate a nuclear plant under different levels of transparency and types of automation. For the condition where transparency was applied at the component level, the operators were required to operate the plant and respond to system upsets. For the condition with plant-wide automation, the operators were required to monitor an agent in operating the plant by itself but intervene in case of interrupts. On a much more limited scale, Guznov et al. (2020) asked their participants to monitor and operate a simple robot in navigating a track. Each time the robot went off-track, the participants were required to intervene and put the robot back on track. Similarly, Sanders et al. (2014) requested their participants to maneuver a soldier through an environment whilst looking for civilians and mark these on a map. In addition, they were asked to assist the soldier’s robotic teammate in responding to navigational requests (i.e., deciding where to go in ambiguous situations). Finally, Chen et al. (2014, 2015) tasked their participants with monitoring UAV and perform manual avoidance maneuvers as a result of hazardous situations in the environment. In addition, a search task was performed where participants marked items of interest at the target area.

In six studies (35%) participants were tasked with only *monitoring the automation*. In Selkowitz et al. (2015), Selkowitz et al. (2017), and Wright et al. (2020), participants were required to monitor an autonomous squad member through a video feed where their primary task was to monitor the actions and information provided by the autonomous squad member. As a secondary task they were asked to monitor the environment for threats. No manual intervention was required for the autonomous squad member. Du et al. (2019) and Pokam et al. (2019) required participants to monitor the behavior of a self-driving vehicle. No intervention was required by the participants irrespective of the scenario or the level of transparency applied. In the study by Panganiban et al. (2020), participants were supported by an automated wingman that was tasked with countering threats by enemy Surface-to-Air missiles. The participants were required to monitor the automation only for the level of support it provided for the mission and the way it communicated its support to the participant.

#### Operationalizations of Transparency and Comparisons

The design of transparent automation depends on the task, the context, and the domain in which the automation is applied. As such, what and how information is displayed to the user is affected by the specific domain in which transparency is applied and what tasks the agent and user are expected to perform. Table 1 provides an overview of the various operationalizations in our sample. As illustration, Selkowitz et al. (2017) and Wright et al. (2020), using the same simulator test-bed, operationalized transparency through displaying icons and colors representing the agent’s status (e.g., a battery indicator), its goals (e.g., a number within an icon representing a way-point on a map), its reasoning (e.g., a time indicator show this as its priority), its projected outcomes (e.g., a red box next to a clock icon indicating a loss of time), and its uncertainty (e.g., a light red border around an event icon). The level of transparency was manipulated by showing more or less of this information per experimental run.

In terms of experimental comparisons, all studies employed a cumulative approach where transparency followed a continuum, that is, from less to more transparent automation. Subsequently, the experiments compared designs with varying levels of transparency and measured their effect on relevant dependent variables. As illustration, Mercado et al. (2016) used the SAT model to develop the user interface for unmanned vehicle operations and designed an experiment to assess the effect of each of the levels of transparency described by the model: Level 1 transparency provided only basic plan information, Level 2 transparency provided the automation’s reasoning and rationale behind the recommendations, and Level 3 provided the automation’s projections and uncertainties. Based on this experimental design, comparisons were made between the levels of transparency in terms of their effect on their dependent variables.

### Describing the Empirical Evidence

Table 2 describes the empirical evidence from each of the studies.

#### Automation Transparency and SA

Situation Awareness was measured in nine out of 17 studies. The instruments that were used to measure the construct were Situation Awareness Global Assessment Technique (SAGAT), Situation Awareness Rating Technique (SART), a confidence in own SA measure, and a Process overview Measure. The results of the studies fell in two categories, improved SA, and no effect.

Selkowitz et al. (2017) reported an effect of transparency on SA in terms of improved L2 and L3 SA when monitoring an autonomous squad member navigating through an urban area. Adding affordances, hazards, environmental constraints, and uncertainties seems to help the operators in obtaining a better picture of the situation. Likewise, Roth et al. (2020) also found improved SA for tasks relating to mission planning and system management for a manned-unmanned helicopter teaming operation. When adding symbols that represented the agent’s reasoning (e.g., the events that justified an intervention), projected outcomes, and uncertainties, improved SA was reported by the participants when using the SAGAT method. Furthermore, Skraaning and Jamieson (2021) found improved SA in their second experiment using SART. Here, nuclear control room operators were given explicit verbal and visual information about automation activities. When verbal feedback was both behavioral and diagnostic (i.e., what equipment failed and why) in contrast to only behavioral or no verbal feedback, operators reported improved SA. Finally, Chen et al. (2014, 2015) reported evidence for the effect of transparency on SA when providing the UAV’s capability to the user. When the UAV provided visual information regarding the changes to its flight path, that is, a presentation of the agent’s previous, present, or projected flight path, SA improved. Likewise, when the operator was able to communicate with the agent using a natural language dialogue (e.g., a message reading “Please control my altitude and speed, I can follow my flight path”) participants reported improved SA.

Some studies found that transparency did not positively affect SA. For example, in their first and third experiment, Skraaning and Jamieson (2021) did not find differences between a traditional and transparent HMI, as measured by SART, when the feedback by the system was limited to behavioral information only (i.e., what equipment failed and not why; first experiment). Furthermore, no effect was found, as measured by the Process Overview Measure, when plant-wide agent-like automation was introduced (third experiment), including detailed information regarding the agent’s historical and ongoing activities. Likewise, Wright et al. (2020) did not find differences in SA between their transparency manipulations. For an autonomous squad member task, they provided in-depth information on the HMI indicating the underlying factors as to why specific surface-level information was presented. However, adding in-depth information did not lead to better SA amongst the participants. Also, Guznov et al. (2020) did not find evidence for improved SA. Participants were tasked with monitoring and controlling a robot through an environment. When the robot communicated its perceptions and actions only (e.g., “I see an obstacle on the right, so I am turning left”), no differences for SA were found compared to when the robot also included its projected future outcomes (i.e., “I see an obstacle on the left, so I will turn right *in order to avoid a collision*”; emphasis added). Moreover, Pokam et al. (2019) found similar results when participants were asked to monitor the actions of an autonomous vehicle. Finally, Selkowitz et al. (2017, 2015) and Roth et al. (2020) did not find an effect of transparency on SA when monitoring an autonomous squad member or when evaluating proposals for an UAV mission (when using the SART method), respectively.

#### Automation Transparency and Mental Workload

Mental workload was measured in two ways: objectively (eye-movements, secondary task performance) and subjectively (NASA-Task Load Index (NASA-TLX), Perceived Task Complexity scale, Dundee Stress State Questionnaire (DSSQ), a 0-100 scale, and the Bedford Mental Workload scale).

First, Selkowitz et al. (2017) used eye-tracking and found that the duration of fixations on the displays increased as a function of transparency. This experiment introduced additional symbology on the display (e.g., motivators for the autonomous squad member, predicted outcomes, uncertainty information), and it appears that adding this information led to increased dwell time on the display. Second, Guznov et al. (2020) also found an increase in mental workload, measured by using the NASA-TLX, as a result of transparency. They found that the primary driver was a significant difference in the “physical workload” sub-scale of the NASA-TLX. The authors concluded that an increase in the amount of text led to additional reading load, which may have been interpreted by the participants as increased physical demand.

Some studies either did not record a difference in mental workload as a function of transparency or recorded a reduction. For experiment 3 in Skraaning and Jamieson’s study (2021), the authors developed two additional displays with which the plant-wide agent-oriented automation could be monitored. These displays showed for example, which part of an automated sequence was being executed, if there were any alerts, the list of actions to be taken, historical and ongoing activities. This information, presented on separate displays, was available in addition to the information in the non-transparent condition. Nevertheless, the operators reported no differences in terms of mental workload. Similarly, Mercado et al. (2016) developed a user interface for evaluating proposed plans for monitoring and controlling multiple unmanned vehicles. Transparency information consisted of text boxes, sprocket graphics, opacity of icons, colors, and bullet points. Mental workload was measured using the NASA-TLX and a range of eye-tracking measures. No differences were found between the transparency levels in terms of mental workload.

Skraaning and Jamieson (2021) measured mental workload using the Perceived Task Complexity scale on nuclear control room operators. In experiment 1 and 2, transparency was introduced at the component-level. That is, transparent automation in this experiment was operationalized in terms of visual presentation of automatic activity next to the components on the displays, dedicated displays for detailed monitoring of controllers and programs and verbal and visual information about the automation’s activities. Providing this additional information resulted in lower perceived mental workload by the participants. For a different task and setting, Panganiban et al. (2020) also found reduced mental workload when an automated wingman communicated its intentions to support the human and to correct the human’s errors. According to this result, knowing that there is an automated teammate present to support one’s actions results in reduced mental effort on the participants’ own tasks. Finally, Chen et al. (2014, 2015) found that providing UAV capability information to the participants resulted in lower workload, as measured by the NASA-TLX.

One study reported that two workload measures were used (NASA-TLX and DSSQ) but did not report the results (Sanders et al., 2014).

#### Automation Transparency and Operator Performance

Operator performance was measured in two ways: objectively (task and response accuracy, response time, detection of events, goal achievement), and subjectively (self-rated task performance). In addition, some studies used more general measures of behavior: verification activities upon receiving advice by the automation, exploration of alternatives and agreement to proposals.

Participants in Mercado et al.’s (2016) study reported improvements in correct acceptances (i.e., an acceptance of a proposal when it was correct) and correct rejections (i.e., a rejection of a proposal when it was incorrect) with increased transparency. Stowers et al. (2020), in a similar study, replicated these results by showing higher percentages of correct responses on proposed plans. Bhaskara et al. (2021) also provided evidence that increased automation transparency leads to improved decision accuracy on proposals provided by an automated agent (“the Recommender”). In terms of response time, Skraaning and Jamieson (2021) found reduced response times for component-level transparency. Transparency focused display design led to faster responses to minor and major systems upsets. In addition, there is some supporting evidence of the positive effect of transparency in terms of faster initiation of evasive maneuvers of UAVs to hazardous events (Chen et al., 2015) and in the time needed to evaluate the validity of planning proposals in a joint helicopter and UAV mission (Roth et al., 2020). Finally, Skraaning and Jamieson (2021) found that increased transparency at the component-level increased detection of process deviations (e.g., alarms) and goal achievement (e.g., successfully executing all steps in a start-up sequence). This result was corroborated by Chen et al. (2015) who found improved goal achievement in terms of items of interest found when performing an UAV search task.

Wright et al. (2020) found little evidence for the effect of transparency on the accuracy of detecting targets in the surrounding environment when monitoring an autonomous squad member. Similarly, Skraaning and Jamieson (2021) reported that when operators were tasked with monitoring plant-wide, agent-like automation performing a cold start-up of a nuclear power plant (experiment 3), no clear benefits were reported when responding to system upsets. In the low transparency condition, the operators had to derive the state of the plant based on process parameters only. In the high transparency condition, the operators had dedicated displays available to show the agent’s plant-wide activities. Still, no differences were found in terms of goal achievement and self-rated task performance. Finally, Mercado et al. (2016) found little evidence for the effect of transparency on response time to planning proposals in an unmanned vehicle military perimeter defense task.

Stowers et al. (2020) reported slower response times to proposed plans made by an intelligent agent when monitoring and controlling multiple unmanned vehicles. Also, Skraaning and Jamieson (2021) reported a reduction in detecting process deviations and in performing verifications of system information when dedicated displays were used showing the activities of the agent-oriented plant-wide automation.

## Discussion

Whilst performing the review, variations in terms of scientific rigor between the studies became apparent. As noted earlier by Bhaskara et al. (2020), experimental studies regarding automation transparency have primarily used non-subject matter experts as participants. It is important that research set in the context of applied-, and safety critical domains, translates to the actual domain it purports to be relevant for. Twelve studies (71%) in our review reported using non-subject matter experts as participants in their experiments. Typically, these studies used university students or laypeople from the local community who were compensated for their effort in terms of course credits or financial payment. Only four studies (23%) used subject matter experts. Skraaning and Jamieson (2021) used licensed nuclear control room operators, Sadler et al. (2016) used airline pilots, and Pokam et al. (2019) and Du et al. (2019) used automobile drivers. One study did not mention what type of participants were used (Guznov et al., 2020). Furthermore, there were large differences in sample sizes between the studies, from 10 to 176 participants. Although more challenging to perform, especially with typically difficult to recruit subject matter experts, studies with larger sample sizes do provide more robust statistical results (Funder & Ozer, 2019; Schönbrodt & Perugini, 2013). This means that the results from some of the studies with relatively small sample sizes should be treated with some caution. Moreover, different studies used different techniques to measure the constructs of SA, mental workload, and operator performance. For example, Roth et al. (2020) measured SA using the SAGAT and the SART method. The SAGAT found a positive effect of automation transparency and the SART did not. Possibly, the SART is more an indicator of confidence in one’s own SA than of SA itself (Endsley, 1988). Nevertheless, comparing results that were based on different measurement methods can be challenging because of differences in sensitivities and reliabilities of these methods. In this study, we have focused on the experimental outcomes, as opposed to the methodological analysis and discussion of the various measurement tools implemented across the reviewed studies.

### Transparency, SA, Mental Workload, and Operator Performance

In the introduction, we alluded to the relationship between SA, mental workload, and operator performance by stating that transparency might alleviate some of the negative effects of automation for SA and operator performance, albeit at the potential cost of mental effort. Increased mental workload arises in cases where multiple tasks are competing for the same resources and task requirements exceed mental capacity (Wickens et al., 2013). When the resources required to build and maintain SA overlap with resources required for task performance, mental capacity may be exceeded which may affect SA and subsequently performance (Endsley, 1995; Endsley & Garland, 2000).

For the relationship between transparency and SA, there are some indications for the increased disclosure of information by an agent and improved SA. Notwithstanding information clutter due to poor interface design (Kim et al., 2011), transparency information may make it easier for an operator to assess what the agent is doing and why by making relevant information readily available to the operator (Endsley, 2017). The studies by Chen et al. (2014, 2015) show overall improvements in SA, the study by Selkowitz et al. (2017) found improved SA for Level 2 and 3 SA (but did not report overall results), and the results from Skraaning and Jamieson (2021) and Roth et al. (2020) show some mixed results depending on how transparency was implemented and which measurement instrument was used respectively. Still, having the information directly perceivable on the interface could reduce the burden on mental processing capacity by reducing the need for keeping multiple information elements in working memory (van Doorn et al., 2021).

For mental workload, only two studies in our sample showed an increase, the remaining studies found either no effect or found a reduction. Interestingly, one of these was measured using eye-tracking and showed an increase in fixation durations, indicating increased information processing with increased transparency (Selkowitz et al., 2017). However, the other study that also measured fixation duration using eye-tracking did not find any significant result (Mercado et al., 2016). Nevertheless, most of our studies seem to indicate that increasing transparency did not affect the participants to such an extent that it led to information overload. Conversely, adding transparency information did not consistently lead to reductions in workload either. In all the experiments in our sample, participants were required to assess the performance of an agent, either through evaluating decision options, intervening in an ongoing process, performing manual activities, or monitoring the agent. One may expect that assessing the performance of an agent, and its associated cognitive effort, would be facilitated when the information about the agent was made available to the user compared to when it was not. Only the studies by Chen et al. (2014, 2015), Skraaning and Jamieson (2021; experiment 1 and 2), and Panganiban et al. (2020) found this effect.

For operator performance, it was expected that performance would improve with increased transparency. There are some indications that transparent automation leads to better discrimination between correct use of proposals and correct rejections in those studies in which this was measured (Bhaskara et al., 2021; Mercado et al., 2016; Stowers et al., 2020). Although some studies did not report any differences in decision accuracy (Guznov et al., 2020; Roth et al., 2020; Wright et al., 2020), there were also no studies that reported a deterioration. This seems to indicate there is some merit in applying transparency principles for tasks where automation usage decisions need to be made. We also found a moderately positive relationship between transparency and response times to events, that is, system prompts or proposals (Bhaskara et al., 2021; Chen et al., 2015; Roth et al., 2020; Skraaning & Jamieson, 2021; experiment 1 and 2).

As good SA, without requiring excessive mental effort, increases the probability for good operator performance (Endsley, 1995; van de Merwe et al., 2012; van Doorn et al., 2021), we assessed those studies in which SA, mental workload, and performance were measured together. Five of the 17 studies measured these three variables in conjunction. For three of these studies, we see neutral or improved SA scores, neutral or reduced workload together with improved response times (Chen et al., 2015; Roth et al., 2020, for SAGAT only; Skraaning & Jamieson, 2021, experiment 1 and 2), goal achievement (Chen et al., 2015; Skraaning & Jamieson, 2021, experiment 1 and 2), and detecting process deviations and performing verifications (Skraaning & Jamieson, 2021, experiment 1 and 2). Guznov et al. (2020) found increased workload scores but no effects for SA and the number of correct responses and correct rejections. Wright et al. (2020) did not find any effect for SA, mental workload, and performance on detecting target and time to identify and assess events. Finally, Skraaning and Jamieson (2021, experiment 3) found no effects for SA, workload, and operator performance, and even reduced performance for detecting and verifying events, when participants were using plant-wide, agent-like automation where transparency information was made available through dedicated displays. This indicates that the benefits of transparency may be affected by agent type, but also how transparency information is made available to operators. The absence of transparency benefits for this study may be attributed to operator capacity issues in simultaneously monitoring the process and the agent, in addition to the attention-grabbing effect of the (separate) transparency interface.

### Transparency and Human-Automation Interaction types

In the results section, we identified that the studies from the sample can be categorized in three distinct human-automation interaction types; that is, participants were tasked with responding to proposals, supervising automation, and monitoring automation. Knowing that the automation interaction paradigm influences system oversight and intervention (Endsley, 2017), a better understanding for which types of tasks transparent automation would provide the most benefit may provide valuable insights to engineers developing transparent designs. The allocation of roles between humans and automation, as well as the automation’s level of sophistication, is important determinants in this relationship (Endsley & Kaber, 1999). For example, automation may provide decision support to a human in direct control (Manzey et al., 2012; Metzger & Parasuraman, 2005; Rieger & Manzey, 2020), or automation may take the form of an intelligent agent that works largely independent, but with the human in a supervisory role, ready to intervene when needed (Borst et al., 2017). As the function distribution between agents and humans dictate the distribution of tasks, this in turn dictates the human information needs to perform these tasks. Different function distributions therefore lead to different operator tasks, which lead to different information (i.e., transparency) needs (van Doorn et al., 2017). Hence, how functions and tasks are distributed between humans and agents is therefore an important element in understanding the relationship between transparency and human performance. As designing collaborative human-agent systems entails making choices with regards to “who does what with what information,” it is important to understand how the purported transparency benefits translate across different human-agent interaction types.

For the studies where participants responded to proposals, the data in Table 2 suggests a relation between transparency and improved correct evaluation of proposals without affecting workload. None of the studies found changes to workload as measured through rating scales, secondary task performance and eye-tracking. For operator performance, the studies by Mercado et al. (2016), Stowers et al. (2020), and Bhaskara et al. (2021) found improved use of correct proposals and improved correct rejection of incorrect proposals. Only Roth et al. (2020) did not find an effect. In terms of response times to proposals however, the picture is less clear. Stowers et al. (2020) found an increase, Mercado et al. (2016) and Bhaskara et al. (2021) found a reduction, and Roth et al. (2020) found no differences. Furthermore, the study by Göritzlehner et al. (2014) showed a reduction in number of separation conflicts, and Sadler et al. (2016) found a reduction in the pilots’ verification of the proposed plans. Unfortunately, there is insufficient data to conclude on SA, as for this interaction type, only one study measured the construct and it showed contrasting outcomes (Roth et al., 2020). Still, the results indicate that transparency has performance benefits for this interaction type without adding workload.

For supervising automation, a moderately positive relation was seen between transparency, improved SA, reduced workload, and improved operator performance. The studies by Chen et al. (2014, 2015) and Skraaning and Jamieson (2021) found no change to SA (experiment 1) or improved SA (experiment 2), reduced mental workload and improved response times, ability to detect events and goal achievement. Skraaning and Jamieson’s third experiment did not replicate these findings. Here they found no differences for SA and workload and a decrease in operator performance. Only Guznov et al. (2020) found an increase in mental workload, when supervising a robot through a maze, with no differences for SA and operator performance reported. Nevertheless, also for this interaction type, the results tend towards performance benefits with limited effect on mental workload.

For monitoring automation, the relationship between the HF variables and transparency is somewhat unclear, however. Only the study by Wright et al. (2020) measured the three constructs for this interaction type but found no differences. None of the other studies captured operator performance, so the data for this construct is rather limited for this interaction type. This is understandable as the participants were not required to do anything other than monitoring. For SA and mental workload, there are some indications for improved SA at the cost of visual processing in monitoring an autonomous squad member (Selkowitz et al., 2017). Reduced mental workload was found when collaborating with an automated wingman (Panganiban et al., 2020). However, the study by Selkowitz et al. (2015) did not find any relationship between transparency, SA, and mental workload. Also, the rest of the (individual) study results did not indicate a relationship with transparency for this interaction type.

### Practical Implications

The results from these studies are relevant for whenever systems are developed where humans are required to work with agents to achieve a common goal. However, the use of agents may provide challenges for human interaction as agents using neural networks are known to be opaque and difficult to interpret (Sanneman & Shah, 2020). As such, although these agents are powerful and flexible in their application, they may come at the cost of interpretability and understandability for a human operator (Doshi-Velez & Kim, 2017). For an agent to be transparent to a human, it would imply the system should provide understandability and predictability of its actions (Endsley, 2017); that is, see into the information processing stages of the agent such that its outcomes are understandable to its user (Hepworth et al., 2020).

Research into strategic conformance, that is, the extent of compatibility between human and agent information processing, seems to suggest improved automation acceptance rates and reduced response times to system proposals. This suggests that systems that “make sense” to the human are easier to supervise as it alleviates some of the workload related to trying to understand what the system is doing and why (Hilburn et al., 2014; Westin et al., 2015). To this end, the well-known human information processing model by Parasuraman et al. (2000) may be used as a basis for developing transparent displays to achieve increased compatibility between human and agent information processing. For example, an agent operating in a real-world setting, for example, an anti-collision tool for autonomous maritime navigation (Statheros et al., 2008), may be able to detect and integrate information based on a suite of sensors, perform object classification, create a representation of its environment, plan actions considering relevant constraints, and execute appropriate actions (DNV, 2018). Making these stages understandable to a user could imply graphically depicting relevant information it has detected (e.g., using bounding boxes around objects), classify this information (e.g., the type of objects and their characteristics), represent their relevance (e.g., in terms of potential collision risks), and indicate potential and highlight optimal solutions based on a cost function (e.g., fuel, time, safety), possibly including uncertainties. Finally, these solutions could be presented as a choice to the operator or automatically executed, depending on the agent’s capabilities.

Adding information to the HMI of an intelligent agent that is compatible with human information processing strategies, provided adequate display design, should imply improved human decision making without adding mental workload. Furthermore, when the human is required to monitor, respond to, and manually operate a function (i.e., supervise), improvements in operator performance, mental workload, and SA can be anticipated when the agent presents the underlying information for its decision making and (proposed) actions. However, careful consideration should be given to how transparency is practically implemented and integrated in existing HMI solutions (i.e., primary task displays) such that operator performance is sufficiently supported (National Academies of Sciences, 2021).

### Limitations

Performing a systematic literature review requires making choices regarding the specificity of the study and its replicability. This review appreciates that there may be research on transparency that is published in non-scientific channels (e.g., reports from research institutes), studies that have researched the construct without using the terms in our search string or have published in channels not captured in our databases. This means that, although this study has aimed to perform a broad review of the literature, it is likely there is research on transparency that is not covered by our SLR. However, for the sake of replicability, this paper has chosen to make the sampling and analysis of the data as objective and open as possible. This means that no additional research was added to the sample that was not found in the search results.

The search spanned a range of over 20 years of research on automation transparency. However, results revealed that experimental studies focusing specifically on automation transparency is a recent topic of interest, at least in terms of number of hits in our data sample. The oldest study in the sample that meets our eligibility criteria was published in 2014. A possible explanation for this are the strict eligibility criteria used. This SLR only includes experimental studies on the topic of transparency, in safety critical domains, for which a limited set of human factors variables were measured. As such, articles that discuss transparency conceptually (e.g., presenting models, frameworks, definitions), that were outside the safety critical domain (e.g., care-giving robots, explainability of algorithms for loan application decisions), that presented secondary data (e.g., reviews), or that did not measure SA, mental workload, or operator performance (e.g., only usability, acceptance, or trust), were not considered. A broader set of eligibility criteria could have resulted in additional data, albeit at the cost of specificity. As such, although transparency has been discussed in publications before (e.g., Endsley et al., 2003; Meister, 1999), there seems to be a relationship between the time the construct was formalized into theoretical models (Chen et al., 2014; Johnson et al., 2014; Lyons, 2013) and the experimental studies these generated.

Finally, differences in statistical reporting made comparison between the studies challenging. Some studies provided full statistical disclosure in terms of *p*-values, effect sizes, confidence intervals, sample sizes, and graphical representations of the data, whereas other studies provided very limited to no statistical information. As such, this made comparison across the studies challenging and prohibited a more rigorous quantitative comparison.

### Conclusions and Future Work

This review mapped the “seeing-into” transparency literature to address the relationships between transparency and central human factors variables. The data provided indications that human performance is enhanced when a function keeps the operator in the loop by presenting proposals and stating the reasons for them. Furthermore, when the human is required to monitor, respond to, and manually operate a function (i.e., supervise), improvements in operator performance, mental workload, and SA can be anticipated when the agent presents the underlying information for its decision making and (proposed) actions. Adding this information to the HMI of an intelligent agent, provided adequate display design, should imply improved human performance without adding mental workload. However, there are subtle variations in SA, mental workload, and operator performance for specific tasks, agent-types, levels of information disclosure, and level of integration of transparency information in primary task displays. Future work should focus on understanding which information types are valuable in conveying agent transparency information (see also National Academies of Sciences, 2021). As a starting point, the information processing model by Parasuraman et al. (2000) was suggested to allow increased compatibility between the agent’s and human’s information processing (Hilburn et al., 2014; Westin et al., 2015). However, the degree to which this model is suitable as tool to set agent transparency requirements should be investigated further.

This study focused on the relationship between agent transparency and operator performance in combination with two primary psychological constructs SA and mental workload. However, automation transparency is frequently researched in relation to other variables, such as trust in automation (Chen et al., 2018; Lee & See, 2004; Oliveira et al., 2020; Schmidt et al., 2020). Trust is the attitude that an agent (or automation) will help achieve a goal in uncertain and vulnerable circumstances (Lee & See, 2004) and is an important element in determining automation usage. Operators may not use automation when they don’t trust it, even though it is reliable. Conversely, high trust in automation may lead to overreliance, that is, using automation when it should not be (Parasuraman & Riley, 1997). Transparent automation should help an operator to calibrate their trust in automation such that automation is only used when it should be (Lee & See, 2004). Although this study did not include trust as part of its inclusion criteria, the relevance of trust in relation to automation transparency is not disputed. Likewise, additional variables such as cognitive processes, system design features, environmental features, and emergent characteristics involved in automation oversight and interaction performance (Endsley, 2017) were similarly excluded. As such, this study focused on the key human variables SA and mental workload in addition to operator performance. Future studies could focus on establishing comprehensive evidence regarding additional key variables in agent transparency and assess their scientific consensus and practical merit.

## Supplemental Material

sj-pdf-1-hfs-10.1177_00187208221077804 - Supplemental Material for Agent Transparency, Situation Awareness, Mental Workload, and Operator Performance: A Systematic Literature Review Click here for additional data file. Supplemental Material, sj-pdf-1-hfs-10.1177_00187208221077804 for Agent Transparency, Situation Awareness, Mental Workload, and Operator Performance: A Systematic Literature Review by Koen van de Merwe, Steven Mallam, and Salman Nazir in Human Factors

## Key Points

• Automation transparency is a design principle aimed at enabling operators to understand what automation is doing, why it is doing it, and what it will do next (i.e., “seeing-into” transparency). It is a means to address the challenges related to human performance in interacting with systems that have high degrees of automation.
• This study systematically gathered and assessed empirical evidence for the relationship between automation transparency, Situation Awareness, mental workload, and operator performance using the PRISMA method.
• There are three transparency models that dominate the transparency research, however, there is a significant body of research investigating transparency without conforming to any particular model. The human-automation interaction types employed in the research can be categorized into responding to agent-generated proposals, supervisory control, and monitoring only. All studies investigated the effect of the amount and type of transparency information on performance variables.
• The empirical results from the studies point towards a promising effect of automation transparency on operator performance, without the cost of added mental workload, for instances where humans respond to agent-generated proposals and where humans have a supervisory role.
• There are subtle variations in SA, mental workload, and operator performance for specific tasks, agent-types, levels of information disclosure, and level of integration of transparency information in primary task displays. There were limited findings for our variables when humans were monitoring automation only.
• The outcomes have practical implications for the design of systems where humans and automation work towards a common goal.
