# Supplementary material for: Agent Transparency, Situation Awareness, Mental Workload, and Operator Performance: A Systematic Literature Review
Source: Hum Factors. 2022 Mar 11;66(1):180–208. doi: 10.1177/00187208221077804 (PMC10756021; doi:10.1177/00187208221077804)
Supplement: sj-pdf-1-hfs-10.1177_00187208221077804 - Supplemental Material for Agent Transparency, Situation Awareness, Mental Workload, and Operator Performance: A Systematic Literature Review [file sj-pdf-1-hfs-10.1177_00187208221077804.pdf]

Reference	Domain	N	Participants	Model	Model description	HAI type	Task description	Operationalization of transparency and comparisons	Situation Awareness	Effect	Mental workload	Effect	Operator performance	Effect
Bhaskara et al., (2021)	Civil UxV	176	Non-SME	1. SAT model	The visualization of the agent's current action and plans (Level 1), its reasoning and constraints (Level 2) and its projected outcomes and uncertainties (Level 3).	1. Respond to proposals	Perform and complete unmanned vehicle control missions by selecting the most appropriate plan (A or B) against mission attributes. Participants were assisted by automation that based its decision on a formula taking into account time to search area, search time and fuel consumption.	Level 1: The automation evaluated each unmanned vehicle's capabilities against the weighted mission attributes to determine and display the most suitable plan. No copy of the automation's formulae was provided.<br><br>Level 1+2: As per level 1. In addition, participants were informed of the automation's formulae and had a hard copy of these.<br><br>Level			Scores on NASA-TLX<br>Secondary task performance (auditory recognition task)	↔<br>↔	Acceptance of correct proposals<br>Rejection of incorrect proposals<br>Accuracy of automation use<br>Decision time	↑<br>↑<br>↑<br>↓

								1+2+3: As per level 1+2. In addition, participants received a visualization of the relative capability projection of the unmanned vehicles associated with Plans A and B (blue shaded bars presented on the interface).					
Göritzlehner et al., (2014)	Air Traffic Control	12	Non-SME	3. Non-specific	Visual representation of a specific automated resolution advisory within the solution space for air traffic (i.e., go or no-go areas in speed and heading for an aircraft).	1. Respond to proposals	Ensure conflict free traffic in a free-flight Air Traffic Control scenario. Respond to resolution advisories by the automation by accepting or rejecting. Rate the agreement with the advisory.	Transparency 1: No support provided. The Solution Space Diagram was turned off.<br><br>Transparency 2: Support provided by showing heading bands, indicating unsafe heading regions.<br><br>Transparen		0-100 scale	↔	Advisory accept/reject<br>Agreement with advisory<br>Separation conflicts<br>Separation violations	↔<br>↔<br>↓<br>↔

								cy 3: Support provided by showing triangle-shaped conflict areas, indicating unsafe regions in speed and heading.					
Mercado et al., (2016)	Military UAV	30	Non-SME	1. SAT model	The visualization of the agent's current action and plans (Level 1), its reasoning and constraints (Level 2) and its projected outcomes and uncertainties (Level 3).	1. Respond to proposals	Monitor and control multiple unmanned vehicles (land, sea, air vehicles) and evaluate proposed plans (A or B) by an intelligent agent based on speed, coverage, and capability.	Level 1: Basic plan information provided by indicating which unmanned vehicles were in use and which paths they used.<br><br>Level 1+2: Level 1 plus the agent's reasoning and rationale behind recommending the plans was provided via a text box and sprocket graphic.<br><br>Level 1+2+3: Level 1+2		Scores on NASA-TLX<br>Mean eye fixation duration<br>Pupil diameter<br>Saccadic amplitude<br>Saccade duration	↔<br>↔<br>↔<br>↔<br>↔	Correct use of proposals<br>Correct rejection of proposals<br>Response time to proposals	↑<br>↑<br>↔

								plus projection of uncertainty information related to a successful outcome. Uncertainty was presented through the opacity of vehicle icons, road colors, sprocket graphic wedges, and bullet points in the text box.			
Roth et al., (2020)	Military UAV	10	Non-SME	1. SAT model	The visualization of the agent’s current action and plans (Level 1), its reasoning and constraints (Level 2) and its projected outcomes and uncertainties (Level 3).	1. Respond to proposals	Perform mission planning and system management of a manned-unmanned teaming operation (manned helicopter + unmanned aerial vehicle). Execute a helicopter transport mission with take-off,	Low transparency: Level 1 information only. For mission planning the automation’s goal, settings, and level of automation were displayed. For system management an “Adopted Tasks”-list was shown.	Scores on SAGAT ↑<br>Scores on SART ↔	Scores on Bedford Mental Workload scale ↔	Response time to proposals ↓<br>Accuracy of decisions ↔

	transit, and landing. In mission planning, the participants had to evaluate the validity of the planning proposals performed by the agent and find violations. In system management, the participants performed the role as pilot-flying in the helicopter. Participants were tasked with monitoring and evaluating the agent's assistance. Flight control were not part of the tasks.	High transparency: Level 1+2+3 information only. For mission planning the automation’s goal, settings, and level of automation (Level 1), symbols representing the events that justified an intervention (Level 2), a timeline presenting the temporal outcomes projected by the agent (Level 3) were shown. For system management an “Adopted Tasks”-list (Level 1), “Critical Events”-list, “Neglected Tasks”-list, and “Current Load”-	

								indicator (Level 2) and "To Do Tasks"-list and a timeline presenting the predicted future workload (Level 3) were shown.			
Sadler et al., (2016)	Flight planning	1 2	SME	3. Non-specific	The provision of the rationale behind automatically derived decision recommendation in three levels of transparency: baseline, value, and logic.	1. Respond to proposals	Land aircraft on a landing site based on recommendations from an Autonomous Constrained Flight Planner.	Baseline: No explanation for how the automation arrived at its recommendation was provided.<br><br>Value: Baseline plus the calculated success probability that drove the diversion recommendation was provided.<br><br>Logic: Logic plus an additional explanation detailing the link between		Verifications of plans Exploring for alternatives Agreement with plans	↓ ↔ ↔

								the probabilities and the information used to derive the recommendations was provided.					
Stower s et al., (2020)	Military UxV	53	Non-SME	1. SAT model	The visualization of the agent's current action and plans (Level 1), its reasoning and constraints (Level 2) and its projected outcomes and uncertainties (Level 3).	1. Respond to proposals	Monitor and control multiple unmanned vehicles (land, sea, air vehicles) and evaluate proposed plans (A or B) by an intelligent agent based on speed, coverage, and capability.<br><br>See also Mercado (2016) in this table.	Level 1+2: Level 1 and 2 information was displayed through the size of the unmanned vehicles' icons with larger icons depicting the faster unmanned vehicles.<br><br>Level 1+2+3: Level 1+2 plus level 3 information displayed by an icon attached to the unmanned vehicles indicating the time it was from its goal location.<br><br>Level 1+2+3+U:		Scores on NASA-TLX	↔	No. of correct responses<br>Response time	↑<br>↑

								Level 1+2+3 plus uncertainty information displayed through changes in opacity of the unmanned vehicles' icons.		
Chen et al., (2014)	Military UAV	43	Non-SME	3. Non-specific	Visualization of the implications of the level of automation on the unmanned aerial vehicle's autonomy capability to the operator through symbols and colors.	2. Supervise automation	Supervise a group of four unmanned aerial vehicles with various functional levels of automation in their search zones. Perform a search operation in the zones.	Non-transparent HMI: The unmanned aerial vehicle did not provide information regarding changes to its current and projected flight path.<br><br>Transparent HMI: The unmanned aerial vehicle provided visual information regarding changes to its flight path.	Scores ↑ on SAGAT	Scores ↓ on NASA-TLX

Chen et al., (2015)	Military UAV	36	Non-SME	3. Non-specific	Visualization of autonomy and functional capabilities of the agent through a (textual) natural language dialogue.	2. Supervise automation	Monitor and control four unmanned aerial vehicles (in various levels of automation) in transit mode, respond to a hazardous event with an avoidance maneuver (hazard avoidance mode) and perform a search activity (search mode).	Limited transparency: No communication mechanism in place to allow the sharing of status between the unmanned aerial vehicles and participant.<br><br>Increased transparency: A message dialogue box was in place to allow communication to be established between the unmanned aerial vehicles and the participant.	Scores on SAGAT ↑	Scores on NASA-TLX ↓	Initial response times for UAV to proceed on-course ↔<br>Event response times for UAV to avoid hazards ↓<br>Success rate in finding items ↑

Guznov et al., (2020)	Robotics	88	Non-SME	1. SAT model	The visualization of the agent's current action and plans (Level 1), its reasoning and constraints (Level 2) and its projected outcomes and uncertainties (Level 3).	2. Supervise automation	Monitor and control a robot through an environment. Based on a video feed and messages from the robot, participants could intervene if the robot was believed to be off the path.	<u>Level 2: The robot's text message informed the participant of its actions and the reasoning processes behind them. For example, when the robot would approach a turn, it would report: "I see an obstacle on the right, so I'm turning left".</u><br><br><u>Level 3: Level 2 plus its future states. For example, when the robot approached a turn, one of the multiple messages it reported was: "I see an obstacle on the left, so I will turn right in order to</u>	Scores on SART	↔	Scores on NASA-TLX	↑	No. of correct responses<br>No. of correct rejections	↔<br>↔

								avoid collision”.			
Sanders et al., (2014)	Ground troops support	73	Non-SME	3. Non-specific	The amount of information that the system provides to the user about its internal operations (i.e., explaining why it behaves as it does).	2. Supervise automation	Control a soldier to find civilians and mark their location on a map. Also, assist an autonomous squad member (i.e., robot) by responding to questions (i.e., make navigational decisions).	Minimal: The autonomous squad member provided a short, three-word description of the problem it encountered.<br><br>Contextual: The autonomous squad member provided the decision it was requesting and a small amount of information about the situation to help the user make the decision.<br><br>Constant: In addition		NASA-TLX results not reported<br>DSSQ results not reported	??

								to the above, the autonomous squad member provided the user with a constant stream of information.						
Skraaning & Jamieson (2021) exp1	Nuclear	16	SME	3. Non-specific	The observability of responsibilities, capabilities, goals, activities, and/or effects of automation in the human system interface.	2. Supervise automation	Control a simulated nuclear power plant and deal with minor to major system upsets including taking corrective action.	Traditional: The human system interface did not provide explicit visual or verbal feedback about automation responsibilities, capabilities, goals, activities, and/or effects. Operator had to infer these attributes of automation from changes in the plant process as reflected in a convention	Scores on SART	↔	Scores on Perceived Task Complexity scale	↓	Response time to events<br>Detecting deviations and performing verifications<br>Achieving main goals	↓<br>↑<br>↑

al human system interface for supervisory control.

Transparent: The interface provided explicit verbal and visual information about automation activities to the operators. Key automatic devices on a large-screen overview display, dedicated displays for detailed monitoring, display of tracking of automation sequences, and verbal feedback about the activity of automatic systems from automation were used.

Verbal feedback

								was provided each time automatic devices started or failed. The information was limited to behavioral feedback from automation (what happened) announced repeatedly.			
Skraaning & Jamieson (2021) exp2	Nuclear	18	SME	3. Non-specific	The observability of responsibilities, capabilities, goals, activities, and/or effects of automation in the human system interface.	2. Supervise automation	Control a simulated nuclear power plant and deal with minor to major system upsets including taking corrective action.	Traditional: The human system interface did not provide explicit visual or verbal feedback about automation responsibilities, capabilities, goals, activities, and/or effects. Operator had to infer these attributes of automation from changes in the plant process as	Scores on SART ↑	Scores on Perceived Task Complexity scale ↓	Response time to events ↓<br>Detecting deviations and performing verifications ↑<br>Achieving main goals ↑

		reflected in a convention al human system interface for supervisor y control.<br><br>Transparen t: The interface provided explicit verbal and visual informatio n about automatio n activities to the operators. Key automatic devices on a large-screen overview display, dedicated displays for detailed monitoring , display of tracking of automatio n sequences, and verbal feedback about the activity of automatic systems from automatio n were used.	

								Verbal feedback was provided only when the executive automatic programs started, or automatic devices failed. The feedback from automation was both behavioral (what happened) and diagnostic (why it happened) and was announced once.						
Skraaning & Jamieson (2021) exp3	Nuclear	27	SME	3. Non-specific	The observability of responsibilities, capabilities, goals, activities, and/or effects of automation in the human system interface.	2. Supervise automation	Control a simulated nuclear power plant and deal with minor to major system upsets including taking corrective action and monitor automatic scripts responsible for performin	Traditional: No explicit information regarding the information about the activities of the plant-wide procedure automation. Operators had to derive this from the process events and	Scores on Process Overview Measure.	↔	Scores on Perceived Task Complexity scale	↔	Detecting deviations and performing verifications<br>Achieving main goals<br>Self-rated task performance	↓<br>↔<br>↔

g a cold start of the plant to 50% reactor power. When the automatic procedure paused (e.g., due to a technical failure) intervene by assuming manual control, restarting automation or shutdown.	changes to system states.<br><br>Transparent: Explicit information about automation status and actions provided through a dedicated overview display showing the automation's progress. Color coding was used to depict status including a detailed list of procedural steps. The detailed automation display depicted historical and ongoing automation activities.

Du et al., (2019)	Autonomous vehicles	32	SME	3. Non-specific	The provision of explanations to justify why an action was or was not taken by the automation.	3. Monitor automation	Monitor the behavior of an autonomous vehicle from the driver's seat. There was no need to take over control of the vehicle.	No explanation: The autonomous vehicle provided no explanation about its actions.<br><br>After explanation: The autonomous vehicle presented an explanation within 1 s after actions had been taken.<br><br>Before explanation: The autonomous vehicle provided an explanation seven seconds prior to its action.		Scores on NASA-TLX	↔

Panganiban et al., (2020)	Military aviation	40	Non-SME	3. Non-specific	The intentional design of a system to communicate its capabilities and current state to support human-machine teaming.	3. Monitor automation	Fly a fighter aircraft and attack a missile site whilst cloaking the plane from detection from a nearby defensive Surface-to-Air-Missile. The autonomous wingman supported the pilot through performing surveillance (neutral condition) or additional cloaking of the pilot against the Surface-to-Air-Missile (benevolent condition).	Neutral: The wingman provided only information on its immediate task activities, with no additional transparency into its intentions.<br><br>Benevolent: The wingman communicated its intention to support the human and to correct its errors, signaling its awareness of the human partner’s expectations.		Scores on NASA-TLX	↓

Pokam et al., (2019)	Autonomous vehicles	45	SME	2. Human-Robot Transparency model	The information that a robot needs to convey to a human (its intentions, tasks, analysis, and environmental constraints) and vice versa (how tasks are distributed and awareness of the human state).	3. Monitor automation	Monitor the behavior of an autonomous vehicle from the driver's seat under different transparency conditions. No intervention was required.	HMI 1: The vehicle displayed no additional information about its autonomy.<br><br>HMI 2: The vehicle displayed its acquired information and its action execution.<br><br>HMI 3: The vehicle displayed its acquired information, its analysis, and its action execution.<br><br>HMI 4: The vehicle displayed its information acquired, its analysis and its decision making.<br><br>HMI 5: The vehicle displayed its information acquired, its analysis,	Scores on SAGAT	↔

								its decision making and its action execution.				
Selkowitz et al., (2015)	Search & Rescue	45	Non-SME	1. SAT model	The visualization of the agent’s current action and plans (Level 1), its reasoning and constraints (Level 2) and its projected outcomes and uncertainties (Level 3).	3. Monitor automation	Monitor an autonomous squad member as it moves through an urban area. The autonomous squad member moved on its own accord taking into account obstacles and dangers. Its route was revealed from waypoint to waypoint by a navigation line.	Level 1: The autonomous squad member provided its current location, its route, and its current resources.<br><br>Level 1+2: Level 1 plus the autonomous squad member provided its affordances and hazards it encounters during its task execution.<br><br>Level 1+2+3: Level 1+2 plus the	Scores on SAGAT	↔	Scores on NASA-TLX	↔

								autonomous squad member provided its environmental constraints with their associated uncertainties and its predicted resources at the end of the mission.				
Selkowitz et al., (2017)	Military UAV	60	Non-SME	1. SAT model	The visualization of the agent's current action and plans (Level 1), its reasoning and constraints (Level 2), its projected outcomes (Level 3), and its uncertainties (Level 3+U).	3. Monitor or automation	Monitor a simulated environment for threats and mark these on the display. Monitor an autonomous squad member's display for its decisions.	Level 1: The autonomous squad member provided its current resource levels, its understanding of the squad's current status, its understanding of the environment around it, the current highest influence on its motivator (e.g., time), and its current action/position.<br><br>Level 1+2:	Scores on SAGAT (L1 SA)<br>Scores on SAGAT (L2 SA)<br>Scores on SAGAT (L3 SA)<br>Confidence in own L1 SA<br>Confidence in own L2 SA<br>Confidence in own L3 SA	↔<br>↑<br>↑<br>↔<br>↑<br>↑	Eye-fixations duration<br>No. of eye fixations<br>Scores on NASA-TLX	↑<br>↔<br>↔

Level 1 plus the autonomous squad member’s reasoning behind its current action (e.g., a clock icon).

Level 1+2+3: Level 1+2 plus the projected outcomes of its current actions and reasoning (e.g., projected time displayed).

Level 1+2+3+U: Level 1+2+3 plus the associated uncertainty for the information (e.g., time uncertainty and icon color).

Wright et al., (2020)	Ground troops support	56	Non-SME	1. SAT model	The visualization of the agent’s current action and plans (Level 1), its reasoning and constraints (Level 2) and its projected outcomes and uncertainties (Level 3).	3. Monitor automation	Monitor a video feed from a soldier team and evaluate the autonomous squad member in correctly identifying and responding to events the soldier squad encountered. Detect threats in the surrounding environment and identify events the squad encountered.	Surface-level information: The interface contained at-a-glance information regarding the autonomous squad member’s current actions (Level 1), the reasons for its action (Level 2) and the results of its actions (Level 3)<br><br>In-depth information: Surface-level information plus additional information depicting the underlying factors that led to each specific information in the surface-level module.	Scores on SAGAT	↔	Scores on NASA-TLX	↔	Detecting targets<br>Time to identify and assess events	↔<br>↔
